# Metabonomic Response to Milk Proteins after a Single Bout of Heavy Resistance Exercise Elucidated by ^1^H Nuclear Magnetic Resonance Spectroscopy 

**DOI:** 10.3390/metabo3010033

**Published:** 2013-01-30

**Authors:** Christian Clement Yde, Ditte Brunn Ditlev, Søren Reitelseder, Hanne Christine Bertram

**Affiliations:** 1 Department of Food Science, Aarhus University, Aarslev, Denmark; E-Mails: ChristianC.Yde@agrsci.dk (C.C.Y.); dittedyt@hotmail.com (D.B.D.); HanneC.Bertram@agrsci.dk (H.C.B); 2 Institute of Sports Medicine Copenhagen, Department of Orthopedic Surgery M, Bispebjerg Hospital and Center for Healthy Aging, Faculty of Health Sciences, University of Copenhagen, Denmark; E-Mail: s.reitelseder@gmail.com (S.R.)

**Keywords:** calcium caseinate, lipoproteins, metabonomics, protein metabolism, muscle hypertrophy, whey

## Abstract

In the present study, proton NMR-based metabonomics was applied on femoral arterial plasma samples collected from young male subjects (milk protein n = 12 in a crossover design; non-caloric control n = 8) at different time intervals (70, 220, 370 min) after heavy resistance training and intake of either a whey or calcium caseinate protein drink in order to elucidate the impact of the protein source on post-exercise metabolism, which is important for muscle hypertrophy. Dynamic changes in the post-exercise plasma metabolite profile consisted of fluctuations in alanine, beta-hydroxybutyrate, branched amino acids, creatine, glucose, glutamine, glutamate, histidine, lipids and tyrosine. In comparison with the intake of a non-caloric drink, the same pattern of changes in low-molecular weight plasma metabolites was found for both whey and caseinate intake. However, the study indicated that whey and caseinate protein intake had a different impact on low-density and very-low-density lipoproteins present in the blood, which may be ascribed to different effects of the two protein sources on the mobilization of lipid resources during energy deficiency. In conclusion, no difference in the effects on low-molecular weight metabolites as measured by proton NMR-based metabonomics was found between the two protein sources.

## 1. Introduction

Muscle hypertrophy occurs when there is a net protein synthesis, and it entails that the muscle synthesis exceeds muscle breakdown. Exercise and, especially, resistance training has a profound effect on muscle protein metabolism [1,2,3], but in the absence of amino acid intake, the balance is negative. The combination of exercise and ingestion of amino acids leads to positive muscle protein synthesis, primarily due to increased muscle protein synthesis. Milk is a valuable source of essential amino acids, and it has been shown that milk protein is an efficient source for muscle build-up [4,5,6].

Casein and whey are the two major protein fractions of bovine milk. Upon intake, micellar casein protein is “slowly” digested, due to its gel formation at the low pH of the stomach, whereas whey protein is digested faster [7,8]. Calcium caseinate digestion and absorption properties probably lie somewhere between those of whey and micellar casein. Thus, studies have shown that intake of whey protein induces a faster postprandial increase in plasma amino acid concentrations compared with intake of casein or caseinate protein [7,9,10]. In addition, it has been shown that the rate of the postprandial insulin response also is faster upon intake of whey protein compared with casein or caseinate protein [10,11,12]. Consequently, these plasma amino acid and insulin analyses have demonstrated that whey and calcium caseinate proteins modulate postprandial metabolism differently. However, it is still not known if these different postprandial responses in plasma amino acid and insulin concentrations are associated with other acute metabolic effects. 

Metabonomics is a tool for detecting various changes in the metabolic profile due to physiological stimuli, changes in food composition and pathological status [13]. The method also provides global insight in physiological processes. What makes metabonomics different from more traditional approaches is the fact that metabonomics is an untargeted and explorative technique aiming at describing the complete metabolic response without any *a priori* knowledge, and metabonomics has gained wide use in biomedical sciences. Also, within nutrition studies, the potential of metabonomics to unravel and depict the metabolic plasma response to a diet have been demonstrated, both in intervention studies performed over several days and in postprandial studies [14,15,16,17,18]. Kirwan *et al.* [19] have investigated the effects of cycling until fatigue by NMR-based metabonomics, however, diet effects were not investigated. Enea *et al.* [20] and Le Moyec *et al.* [21] investigated metabolome changes after exercise. Furthermore, studies on exercise-induced metabolic changes have been carried out using GC-MS on plasma, and the data have been elucidated by a metabonomic approach [22,23]. Nevertheless, metabonomic investigations of postprandial responses in combination with exercise are sparse [24]. Since physical exercise will inevitably require nutrition and energy, elevate the metabolism and generate more metabolic products, the level of endogenous metabolites will change accordingly, and metabonomic studies on plasma samples could be expected to gain important insight into exercise-induced changes in metabolism and the impact of nutrition source during the post-exercise period.

Consequently, the aim of the present study was to investigate the postprandial metabolic changes to intake of milk protein drinks (whey or calcium caseinate) in combination with heavy resistance training in order to elucidate potential differences in the postprandial response to the two different protein sources. We therefore present a proton NMR-based metabonomics procedure applied on femoral arterial samples collected from young male subjects at different time intervals after heavy resistance training and intake of either whey or calcium caseinate protein.

## 2. Results

### 2.1. Post-Exercise Effect

**Figure 1:**
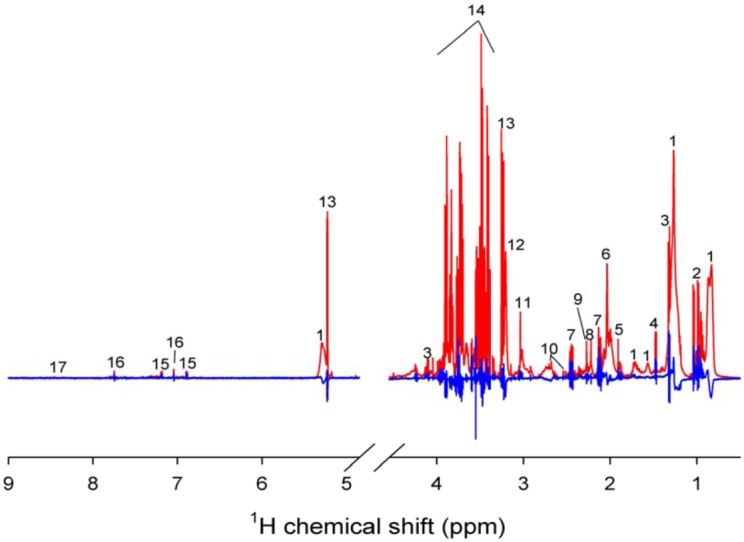
Representative 600 MHz ^1^H nuclear magnetic resonance (NMR) spectrum of a plasma sample 70 min after a bout of heavy resistance and intake of protein drink (calcium caseinate) (Red line). The difference between the 70 min post-exercise sample and the corresponding -70 min pre-exercise sample from the same individual is also shown (Blue line). Nomenclature: 1, lipid; 2, branched amino acids; 3, lactate; 4, alanine; 5, acetate; 6, N-acetyl glycoprotein; 7, glutamate/glutamine; 8, acetone; 9, acetoacetate; 10, citrate; 11, creatine; 12, cholines; 13, glucose; 14, mainly glucose region; 15, tyrosine; 16, histidine; 17, formate.

A representative 600 MHz ^1^H NMR femoral arterial plasma spectrum obtained from a subject at 70 min after both heavy resistance and intake of protein drink (calcium caseinate) is shown in Figure 1. 

**Figure 2:**
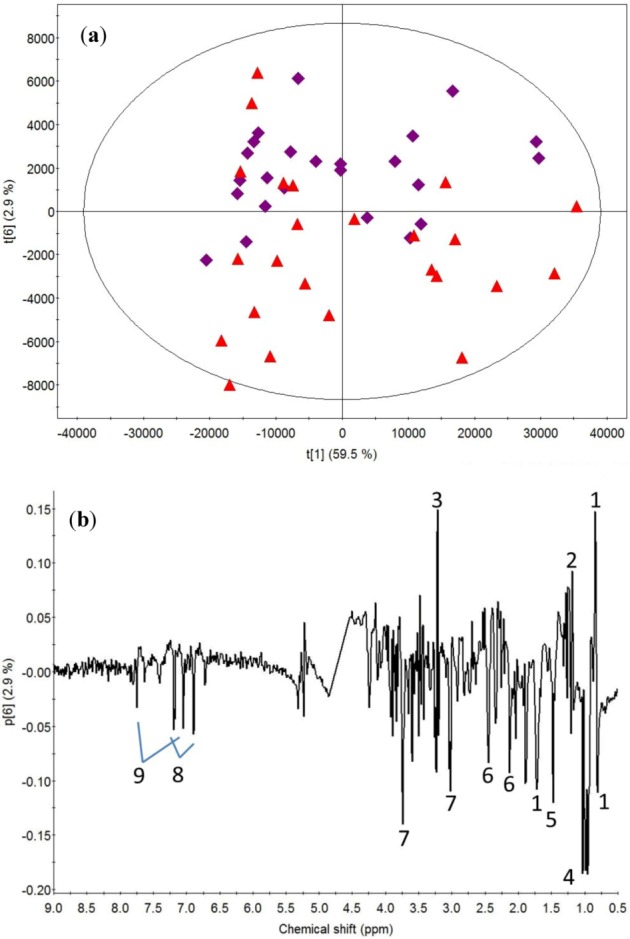
(**a**) Principal component analysis (PCA) score plot of all ^1^H NMR data from blood samples taken -70 (violet-diamond) and 70 (red-triangle) minutes after a bout of heavy resistance training. (**b**) PCA loadings for component 6. Nomenclature: 1, lipid; 2, beta-hydroxybutyrate; 3, choline; 4, branched amino acids; 5, alanine; 6, glutamate/glutamine; 7, lysine; 8, tyrosine; 16, histidine. The ellipse represents the Hotelling T2 with 95% confidence.

To determine the effect of exercise, PCA was carried out on all -70 and 70 min samples (Figure 2). Principal component (PC) 6 in the score plot discriminates between the pre- and post-exercise samples. The loadings ascribe these changes to a decreased intensity of beta-hydroxybutyrate and choline and an increased intensity of amino acids: alanine, branched amino acids, glutamate, glutamine, histidine, lysine and tyrosine after exercise. No clear effects of exercise were observed on the signals from lipids.

**Figure 3:**
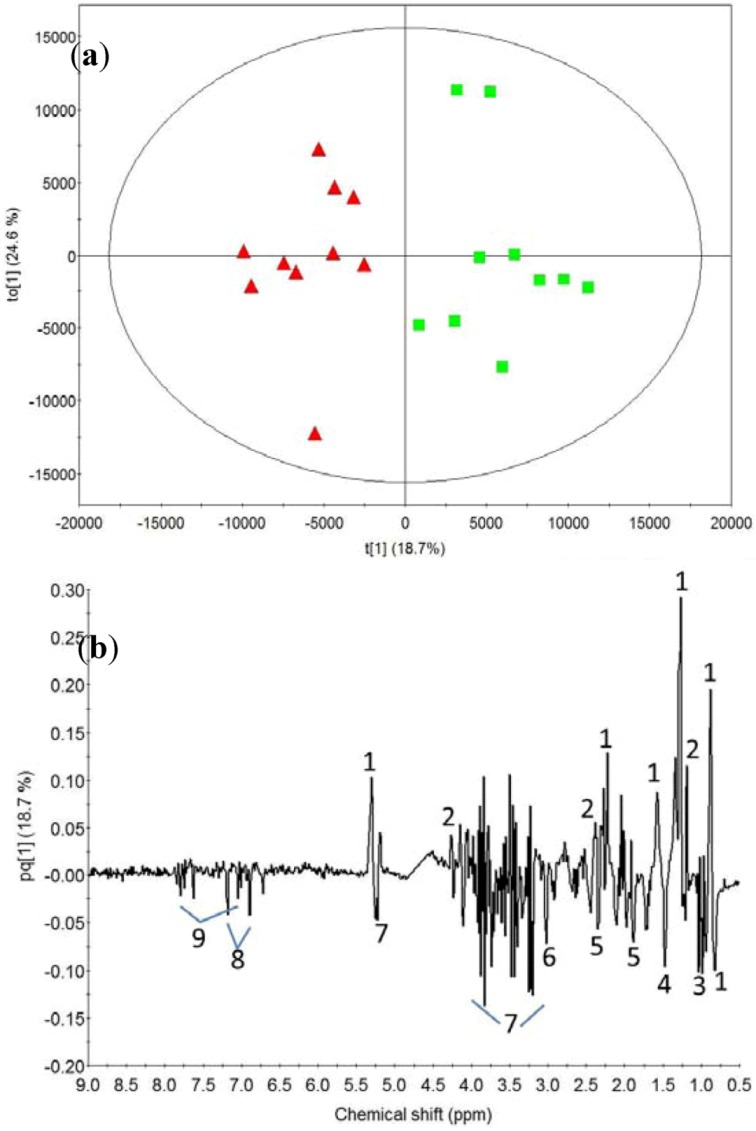
(**a**) Orthogonal partial least squares regression-discriminate analysis (OPLS-DA) score plot of baseline subtracted ^1^H NMR data from blood samples taken 70 (red-triangle) and 220 (green-box) minutes after a bout of heavy resistance training and the intake of calcium caseinate drink. (**b**) PCA loadings for component 5. Nomenclature: 1, lipid; 2, beta-hydroxy butyrate; 3, branched amino acids; 4, alanine; 5, glutamate/glutamine; 6, creatine; 7, glucose; 8, tyrosine; 9, histidine. The ellipse represents the Hotelling T2 with 95% confidence.

PCA was performed on the baseline-subtracted femoral arterial plasma samples for both whey protein and calcium caseinate. Both treatments showed a separation between the blood samples taken at 70 min after a bout of heavy resistance and the later time points (220 and 370 min; data not shown). To explore the biochemical differences, Figure 3 shows an OPLS-DA model with one predicted and one orthogonal component (R2X = 0.42, R2Y = 0.82 and Q2 = 0.53) between time-points 70 and 220 min for the calcium caseinate treatment. The loadings for the predicted component indicates a higher intensity of beta-hydroxybutyrate and lipids at 220 min and a higher intensity of alanine, branched amino acids, creatine, glucose, glutamate/glutamine, histidine and tyrosine at 70 min after resistance exercise (Figure 3(**b**)). A similar OPLS-DA model for the intake of whey protein revealed similar dynamic changes in the post-exercise period (data not shown). 

### 2.2. Milk Protein Effect

**Figure 4:**
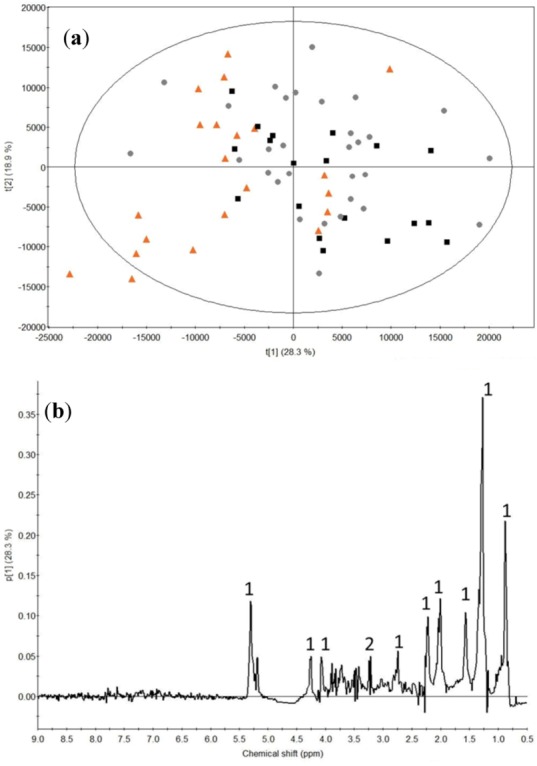
(**a**) PCA score plot of all baseline subtracted plasma samples showing the different protein sources: orange-triangle, control; black-box, whey protein; grey-dot, calcium caseinate protein. (**b**) PCA loadings for component 1. Nomenclature: 1, lipid; 2, choline. The ellipse represents the Hotelling T2 with 95% confidence.

To examine the differences between the different drinks ingested, PCA was carried out on all the baseline-subtracted femoral arterial plasma samples (Figure 4). The control samples are found to separate along PC 1 from the whey and calcium caseinate samples. This discrimination can be ascribed to higher intensities of lipids and choline-containing compounds (choline, phosphocholine and glycerophosphocholine) after intake of milk proteins compared to the control. In addition, PCA models obtained at the individual time points for the milk protein samples discriminated between whey and calcium caseinate at 70 min after a bout of heavy resistance training. The loadings indicated higher lipid signal intensity from 0.81-0.85 ppm and 1.22-1.27 ppm (data not shown).

**Figure 5:**
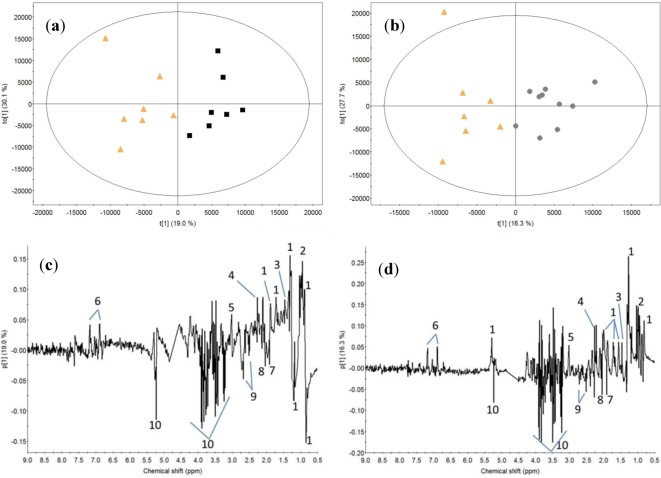
OPLS-DA score plots discriminating between control and whey (**a**) and calcium caseinate (**b**) at 70 min (baseline subtracted) after a bout of heavy resistance. Corresponding loading plot describing the component for OPLS-DA models of control *vs.* whey (**c**) and control *vs.* calcium caseinate (**d**). Nomenclature: 1, lipid; 2, branched amino acids; 3, alanine; 4, acetoacetate; 5, creatine; 6, tyrosine; 7, acetate; 8, N-acetyl glycoprotein; 9, citrate; 10, glucose. The ellipse represents the Hotelling T2 with 95% confidence.

Subsequently, two OPLS-DA models with one predicted and one orthogonal component were performed to elucidate differences between the control samples and whey or calcium caseinate samples, respectively, at time point 70 min for baseline subtracted data (Figure 5; water *vs.* whey: R2X = 0.49, R2Y = 0.81 and Q2 = 0.59; water *vs.* calcium caseinate: R2X = 0.44, R2Y = 0.79 and Q2 = 0.52). The loadings for the predictive components of both models show that intake of both whey and calcium caseinate proteins results in a higher intensity of branched amino acids, alanine, acetoacetate, creatine and tyrosine and a lower intensity of acetate, glycoproteins, citrate and glucose. However, after intake of calcium caseinate protein, all the lipid signals have positive loading values, whereas the lipid signals for the difference between non-caloric and whey drinks have both positive and negative values in the OPLS-DA loadings. The OPLS-DA model discriminating between control and whey (Figure 5 (**b**)) points to the higher intensity of the lipid signals from CH3 groups at 0.80-0.86 ppm and CH2 groups at 1.18-1.28 ppm. Conversely, the lipid signals at 0.87-0.90 ppm and 1.28-1.36 ppm have decreased intensity for whey samples compared to control samples. 

## 3. Discussion

### 3.1. Post-Exercise Effect

In the present study, we have employed an NMR-based metabonomic approach to investigate the physiological processes and dynamic metabolic changes in femoral arterial plasma samples as a result of a bout of heavy resistance training in combination with intake of whey or calcium caseinate protein drinks. The post-exercise time-effect revealed that the femoral arterial blood differentiated 70 min after exercise and treatment from 220 and 370 min. Standard amino acids analyses performed on samples from the present study have shown that the plasma concentration of various amino acids peaked 45 minutes after intake of the protein drink and returned to baseline 210-360 minutes after intake [10]. Thus, the higher amino acid intensities for the 70 min samples than the baseline and the later time points measured by ^1^H NMR-based metabonomics are in good agreement with the standard analyses. The higher plasma beta-hydroxybutyrate concentration prior to exercise (fasting) and the later post-exercise effect could explain the production of ketone bodies by fat oxidation. In addition, no dynamic differences could be detected between the intake of a whey protein or a calcium caseinate drink after heavy resistance training. Measurement of a faster postprandial increase in plasma amino acid concentrations associated with the intake of whey compared to calcium caseinate would probably require sampling prior to the 70 min sampling, which was the first time point included in the present study. 

### 3.2. Milk Protein Effect

Studying the difference between the post-exercise period by the three sampling points at 70, 220 and 370 min after exercise indicated that the overall effect of intake of milk protein (whey or calcium caseinate) compared to a non-caloric drink is higher levels of lipids and cholines in the blood. This finding reflects the increased content of lipoproteins after absorption of nutrients in the gastrointestinal tract upon intake of whey or calcium caseinate protein drinks. OPLS-DA models with whey and calcium caseinate as classes made to investigate the difference between whey and calcium caseinate intake were poor, and they were unsuitable for interpretation (Q2 < 0.5; data not shown). Thus, to investigate only the short-term influence of intake of either whey or calcium caseinate protein drinks after a single bout of heavy resistance, OPLS-DA models were performed in comparison with the intake of the non-caloric drink at 70 min after intake. In comparison with the non-caloric drink, both whey and calcium caseinate protein drink intake revealed differences in the lipid signals mainly assigned to either LDL (0.87-0.90 and 1.28-1.36 ppm) or VLDL (0.80-0.86 and 1.18-1.28 ppm) [25,26]. Consequently, the present study indicates that whey protein increased VLDL and decreased LDL lipoprotein, whereas calcium caseinate protein increased both VLDL and LDL lipoprotein content in the plasma. The results were obtained with the CPMG pulse sequence [26], which attenuates the lipid signals; single-pulse spectra are better suited for assessing the protein-source effects on the lipoproteins. In-depth investigations of the lipoprotein changes can be achieved by ^1^H NMR spectroscopic methods described by Ala-Korpela *et al.* [28,29]. However, that is outside the scope of the present study. 

The earlier onset of an increase in the amino acid concentration in the whey-treated group may be due to the faster digestion of the protein and, thereby, a higher maximum amino acid concentration in the blood. It could be expected that this difference in amino acid concentration observed about 15-45 min after exercise for the whey and calcium caseinate groups was accompanied by other metabolic effects. However, the present examination of the blood metabolome at 70, 220 and 330 min after exercise and protein intake showed a similar change in the blood metabolome for the whey and calcium caseinate protein treatment groups. Despite the differences in the amino acid composition of the whey and calcium caseinate protein drinks, both whey and calcium caseinate protein drink intake had similar effects on the level of low-molecular weight metabolites in plasma when compared with the non-caloric drink. This finding could seem surprising, but should probably be explained by the fact that differences in the uptake of amino acids between the two protein sources, as seen previously [10], set in earlier than the first time point at 70 min. Even though no profound effect of protein source on the blood metabolite profile was found, it is expected that the present approach could be useful for elucidating more severe effects related to differences in the degree of exercise and/or energy source and energy intake. 

## 4. Experimental Section

### 4.1. Subjects

A full description of the study is given in Reitelseder *et al.* [10]. Twelve healthy male subjects were randomized to participate in two protein trials in randomized order (three individuals only participated in the first period, whey protein n = 2, calcium caseinate protein n = 1). Eight male subjects participated in a control trial. Mean ± S.E.M. for control and milk protein group, respectively: age: 26 ± 2, 28 ± 2 y; weight: 74 ± 2, 79 ± 3 kg; body mass index (BMI): 22.7 ± 0.7, 24.3 ± 0.7 kg∙m-2; lean body mass (LBM): 57 ± 2, 58 ± 2 kg; one repetition maximum (1RM; *i.e.* is the maximum amount of weight one can lift in a single repetition for a given exercise): 67 ± 4, 66 ± 4 kg. All participants were recruited with the criteria of being moderately active young males with no history of regular participation in aerobic or resistance training during the last 6 months. All participants were non-smokers and on a normal western diet adequate with regards to protein content (minimum of 0.8 g ∙ kg-1 ∙ d-1). Additionally, no family histories of diabetes or chronic medication were allowed. Before inclusion, study design, purpose and possible risks were explained to each subject, and subsequently, all subjects gave their written consent to participate in the protocol, which adhered to the Helsinki declaration and was approved by the local Ethics Committee of Copenhagen and Frederiksberg (H-KF 2007-0014). 

### 4.2. Pre-Tests and Food Registration

The pre-tests were performed on two separate days. The aim of the first day was to familiarize the subjects to test equipment and the protocol, and subsequently, on the second day, the determination of 1RM was conducted. The two test days were separated by at least one week, and the 1RM determination was at least two weeks prior to the experiment. All subjects were instructed not to perform any strenuous activity during the three pre-experiment days.

### 4.3. Experimental Protocol

**Scheme 1:**
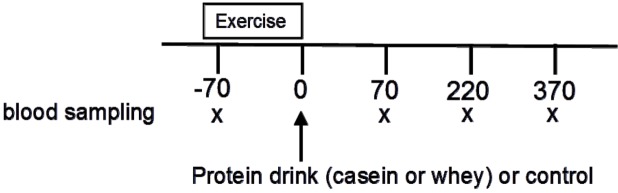
Experimental protocol. A single bout of heavy resistance exercise was performed following intake of test or control drink. Femoral arterial blood samples were collected through the protocol.

The experiment was conducted as outlined in Scheme 1. All subjects arrived to the laboratory by car at 7:00 a.m. after an overnight fast (10 hours). An antecubital venflon was inserted and a background sample obtained. The femoral artery of the exercise leg was cannulated under local anesthetic treatment (lidocaine, 1%). Applying the Seldinger technique, 20 gauge catheters (Arrow, ES-04150, Reading, PA, USA) were inserted and kept patent with NaCl during the experiments. On the arterial side, a pressure bag (VBM Medizintechnik, Sulz a.N., Germany) was inflated to maintain a pressure of ~200 mmHg during the saline infusions. Catheters were secured with sutures, and the sites of insertion were frequently observed throughout the experiment. In 5 out of 29 experiments (whey: n=3; calcium caseinate:n=1; control: n=1) out of total 29 experiments (21 milk protein and 8 control experiments) it was not possible to insert the arterial catheter, and no femoral artery samples were obtained for these experiments. The acute heavy resistance exercise bout consisted of 10 sets with 8 repetitions at a pre-determined load corresponding to 80% of 1RM. In between sets were rest periods of 3 min. The exercise protocol aimed at stimulating the quadriceps muscle maximally. The chosen exercise was a one-legged, seated leg-extension (Technogym, Super Executive Line, Gambottola, Italy) with a range of motion from 100 to 30 degrees. Immediately after completion of the exercise, between time -60 and 0 min, the participants received a drink containing either water, whey protein isolate or calcium caseinate at time 0 min (for amino acid composition, see Reitelseder *et al.* [10]. All subjects were blinded with regard to what drink they were receiving. The amount of protein was adjusted to 0.30 g • kg lean body mass (LBM)-1 and was dissolved in ~400 mL of water. This dose was chosen, because it corresponded to approximately 20 g of proteins, however, taking into account the individual LBM of the subjects. The drinks were consumed within ~5 min. Blood sampling was collected during pre-exercise at -70 min (baseline) prior to the end of heavy exercise resistance and post-exercise at 70, 220 and 370 min after heavy exercise resistance.

### 4.4. NMR Measurements

An amount of 400 μL aliquots of the plasma samples were mixed with 200 μL of D2O. The NMR measurements were performed at 310 K on a Bruker Avance III 600 spectrometer, operating at a ^1^H frequency of 600.13 MHz, and equipped with a 5 mm ^1^H TXI probe (Bruker BioSpin, Rheinstetten, Germany). A Carr-Purcell-Meiboom-Gill (CPMG) pulse sequence with water suppression was applied for acquisition of ^1^H NMR spectra, with the CPMG delay added to attenuate signals from macromolecules. The total spin–spin relaxation delay was 100 ms, the spin-echo delay was 1ms and the recycle delay was 2 s. The spectra were acquired by 64 scans into 32 k data points on a spectral width of 17.34 ppm. A fixed receiver–gain value was used for recording all samples. An exponential line-broadening of 0.3 Hz was applied prior to the Fourier transformation. Each spectrum was manually phased, baseline-corrected and referenced to the anomeric α-glucose doublet signal at 5.23 ppm. The NMR signals have been tentatively assigned to various metabolites based on existing literature [30] and the Human metabolome database [31]. The region between 0.91-1.05 containing signals from isoleucine, leucine and valine is characterized as the branched amino acid region. 

### 4.5. Data Handling

The region at 0.5–9.0 ppm of the ^1^H NMR spectra were segmented into bins of 0.01 ppm and integrated. To exclude the water signal, the region at 4.55–4.85 ppm was not included in the further multivariate data analysis. The unsupervised method principle component analysis (PCA) was performed on Pareto-scaled and mean-centered binned NMR data on all the samples to detect clustering behavior and explore biochemical differences. In addition, orthogonal partial least squares regression-discriminate analysis (OPLS-DA) was carried out on pre-defined classes. The quality of the models was evaluated by: R2X describing how much of the variation that is explained by the model (goodness of fit) and Q2 representing the predictive ability of the model (goodness of prediction). All models were validated by full cross-validation. The multivariate data analysis was carried out using the SIMCA-P+ version 12.0.1.0 (Umetrics, Umeå, Sweden). In order to eliminate the inter-subject variation, ^1^H NMR spectra obtained on plasma samples collected prior to exercise and protein intake (baseline spectra) were subtracted from the ^1^H NMR spectra obtained on plasma samples collected at later time points. This approach has previously been described by Bro and Smilde [32] and applied by Yde *et al.* [33]. 

## 5. Conclusions

The present study investigated the potential of NMR-based metabonomics for elucidating the postprandial metabolic changes to intake of whey or calcium caseinate protein drinks in combination with heavy resistance training. The approach enabled us to study the dynamic metabolic changes in the metabolite profile after exercise and protein intake. Overall, there is no effect in small metabolites between the two protein sources, as measured by ^1^H NMR-based metabonomics, when examining the plasma profiles. 
